# Advantages of Skeletal Muscle Preservation in Settings of Weight Loss

**DOI:** 10.1111/acel.70521

**Published:** 2026-05-07

**Authors:** David J. Glass

**Affiliations:** ^1^ Regeneron Pharmaceuticals Tarrytown New York USA

## Abstract

There has been a dramatic increase in the use of GLP1R agonists and related “incretins” to treat individuals diagnosed with obesity. However, it has only been recently highlighted that, in addition to the desired loss of adiposity, these medicines also cause a loss of skeletal muscle. It has been debated whether this loss is an issue. Here the consequences of that loss are discussed, and the population most at risk is highlighted. Also, it is pointed out that there are metabolic benefits to maintaining skeletal muscle mass—including a heightened and desirable further loss of adiposity.

## Graphical abstract

“Quality weight loss” can be distinguished from regular weight loss in that the skeletal muscle is preserved. This can be achieved pharmacologically, by inducing mechanisms that preserve or increase skeletal muscle. Created in BioRender. Egerman, M. (2026) https://BioRender.com/88ucpur.

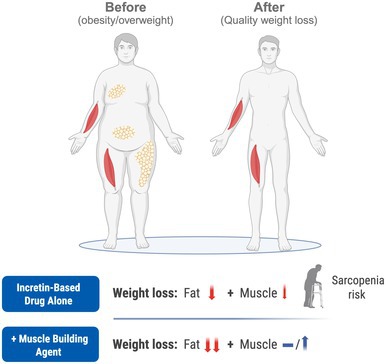

Over the last several years, there has been a dramatic inc rease in the use of GLP1R agonists and related “incretins” to treat individuals diagnosed with obesity. By some estimates, 1 out of 7 adults in the United States have already taken a GLP 1R agonist and one in eight of all Americans reported being currently on a GLP1R agonist this past November (Poll 2025). These medicines act at multiple sites within the body to delay gastric emptying and inhibit food intake, including both centrally (at the hypothalamus and hindbrain; Gabery et al. 2020) and peripherally (via inhibition of gastric emptying; Hjerpsted et al. 2018). By restricting caloric intake, GLP1R agonists restrict caloric intake, allowing for weight loss.

Of interest, though, is the fact that a substantial fraction of GLP1R‐associated weight loss is lean mass—with estimates suggesting that roughly one third to one half of that lean mass may reflect loss of skeletal muscle (McCrimmon et al. 2020; Prado et al. 2024; Sargeant et al. 2019). This loss of skeletal muscle is not unique to settings where GLP1R agonists are used; lean mass loss is a common observation across interventions that drive rapid, robust weight loss (intensive(?) diet and lifestyle intervention, bariatric surgery, etc.). Still, because obesity is defined as excess adiposity—not simply excess body weight—the loss of skeletal muscle may be an undesirable tradeoff in certain populations.

In younger adults, this loss of skeletal muscle may not be a major issue. Younger adults with obesity put on the skeletal muscle required to carry their higher body weight—so it shouldn't be a concern if they lose that muscle when they drop weight while either on a diet or during use of a medication which induces or facilitates dieting behavior. Furthermore, if a younger adult regains weight after discontinuing medication, they would generally be expected to also regain the skeletal muscle needed to support that weight (Hall 2007; Ten Hoor et al. 2018; Tomlinson et al. 2016).

It's a different story for elderly adults, however. In older individuals, muscle gain is more difficult. With age, a number of mechanisms are induced that blunt muscle anabolism, including increased chronic inflammatory signaling, a decline in mitochondrial gene expression, and “IGF1 insensitivity.” (Egerman and Glass 2014; Shavlakadze et al. 2023) These factors likely contribute to age‐related loss of muscle mass and function—sarcopenia—which is now recognized as a disease associated with loss of mobility, frailty, and ultimately increased mortality (Rooks and Roubenoff 2019; Roubenoff and Hughes 2000).

Therefore, if an older person comes off a GLP1R agonist and regains weight, the concern is that adiposity may return more readily than muscle. In that scenario, the individual may be left carrying a higher body weight with more fat and with less skeletal muscle required to carry that overall weight, potentially accelerating the trajectory toward sarcopenia (Wilding et al. 2022; Linge et al. 2024; Sargeant et al. 2019; Prokopidis, Daly, and Suetta 2025; Prokopidis, Tessier, et al. 2025). Consistent with this concern, some studies—particularly in post‐menopausal women—have shown that GLP1R‐associated lean mass loss can translate into clinically meaningful reductions in strength and frailty‐related outcomes (Prokopidis, Daly, and Suetta 2025).

Given the concerns raised by the demonstration of lean mass loss while on GLP1R agonists, there has been renewed interest in preserving skeletal muscle during medically‐induced weight loss by inhibiting negative regulators of muscle growth, namely myostatin and related ligands—such as ActivinA—or by blocking their shared ActRII receptors. This approach was recently tested in rodents and non‐human primates (Mastaitis et al. 2025). In these experiments, GLP1R agonists promoted both fat and skeletal muscle loss, while co‐treatment—either with a neutralizing antibody to ActRII receptors or with antibodies blocking myostatin ± Activin A—not only maintained (and in some cases increased) skeletal muscle mass, but also markedly increased fat loss (Mastaitis et al. 2025). Furthermore, in a study of post‐menopausal women, it was shown that combining anti‐myostatin and anti‐Activin A antibodies led to enhanced muscle growth in comparison to anti‐myostatin treatment alone—and to reductions in adiposity (Gonzalez Trotter et al. 2025).

In light of these data, it is worth contemplating longer‐term myostatin or ActRII pathway inhibition in humans, especially in elderly or frail individuals living with obesity who are treated with GLP1R agonists. One way to better understand the health consequences of prolonged inhibition in humans is to study the phenotype of individuals with naturally occurring *MSTN* variants that reduce or eliminate gene function. These individuals have experienced a lifelong genetic dampening of the pathway. Individuals with *MSTN* mutations gleaned from a large multi‐cohort dataset (with exome sequencing and detailed anthropometric phenotypes) were studied; it was found that individuals with such mutations in general have more skeletal muscle mass than is normally found in their age groups, less adiposity, and greater strength (as determined by grip strength measurements) (Herman et al. 2026). It is reassuring to learn that individuals with lifelong decreases in myostatin signaling appear not only to tolerate such a condition but benefit from it. It should be noted, however, that pharmacological inhibition of myostatin or myostatin receptor signaling hasn't reliably translated into improved function. This might be due to the clinical outcome measures used, or it may indicate that more work needs to be done to understand how function can be improved. Clearly, though, clinical trials need to assess functional outcomes—mobility, strength and avoidance of disability—in a convincing manner.

Completely distinct from the importance of maintaining skeletal muscle to avoid weakness and frailty, it's compelling to learn that when anti‐myostatin and anti‐activin A antibodies are studied in combination with a GLP1R agonist, the combined approaches are associated with an almost doubling of adiposity loss in mice, while maintaining or even increasing skeletal muscle mass (Mastaitis et al. 2025). This effect may reflect a simple (and appealing) physiology: preserving a larger pool of metabolically active tissue—skeletal muscle—during caloric restriction may force greater reliance on fat stores, improving the *composition* of weight loss rather than simply the magnitude of weight loss.

Since obesity is a disease of adiposity in particular, therapies that further drive fat loss while maintaining skeletal muscle seem to be very attractive, especially in populations at higher risk of sarcopenia, in comparison to therapies that are simply focused on weight loss, without concern for the “quality” of that weight loss. Since the population is aging, the prevalence of sarcopenia will be expected to increase in the years ahead. The loss of skeletal muscle with age leads to frailty and loss of independence—the inability to carry out the normal tasks of independent living. It would be a significant public health concern indeed if current weight‐loss strategies increased the risk of such outcomes (Rooks and Roubenoff 2019; Roubenoff and Hughes 2000).

## Author Contributions

D.J.G. conceived and authored this perspective piece.

## Funding

The author has nothing to report.

## Conflicts of Interest

D.J.G. is an employee of Regeneron and owns stock and stock options in the company.

## Data Availability

As this is a perspective piece, no original data was provided in this manuscript so there is no need to specify data availability.
